# Voluntary running-induced activation of ventral hippocampal GABAergic interneurons contributes to exercise-induced hypoalgesia in neuropathic pain model mice

**DOI:** 10.1038/s41598-023-29849-6

**Published:** 2023-02-14

**Authors:** Kohei Minami, Katsuya Kami, Yukihide Nishimura, Makoto Kawanishi, Kyosuke Imashiro, Takuma Kami, Shogo Habata, Emiko Senba, Yasunori Umemoto, Fumihiro Tajima

**Affiliations:** 1 Department of Rehabilitation Medicine, Wakayama Medical University, Wakayama, Japan; 2 Department of Rehabilitation, Faculty of Wakayama Health Care Sciences, Takarazuka University of Medical and Health Care, Wakayama, Japan; 3 Department of Rehabilitation Medicine, Iwate Medical University, Morioka, Japan; 4 Department of Physical Therapy, Osaka Yukioka College of Health Science, Ibaraki, Japan

**Keywords:** Neuroscience, Molecular neuroscience, Experimental models of disease, Pain management

## Abstract

The exact mechanism of exercise-induced hypoalgesia (EIH) in exercise therapy to improve chronic pain has not been fully clarified. Recent studies have suggested the importance of the ventral hippocampus (vHPC) in inducing chronic pain. We investigated the effects of voluntary running (VR) on FosB^+^ cells and GABAergic interneurons (parvalbumin-positive [PV^+^] and somatostatin-positive [SOM^+^]) in the vHPC-CA1 in neuropathic pain (NPP) model mice. VR significantly improved thermal hyperalgesia in the NPP model. The number of the FosB^+^ cells was significantly higher in partial sciatic nerve ligation-sedentary mice than in Sham and Naive mice, whereas VR significantly suppressed the FosB^+^ cells in the vHPC-CA1. Furthermore, VR significantly increased the proportion of activated PV^+^ and SOM^+^ interneurons in the vHPC-CA1, and tracer experiments indicated that approximately 24% of neurons projecting from the vHPC-CA1 to the basolateral nucleus of amygdala were activated in NPP mice. These results indicate that feedforward suppression of the activated neurons via VR-induced activation of GABAergic interneurons in the vHPC-CA1 may be a mechanism to produce EIH effects, and suggested that disappearance of negative emotions such as fear and anxiety by VR may play a critical role in improving chronic pain.

## Introduction

Chronic pain is a major health problem that affects 11–40% of people worldwide^1^. In Japan, 15% of patients suffer from chronic pain^2^. Additionally, deterioration in quality of life (QOL) due to pain results in a decline in work productivity, and the rising cost of medical care for pain treatment is considered an important social problem^3,4^. Clinical trials and guidelines recommend a personalized, complex, multidisciplinary treatment approach that includes medication, exercise, and psychotherapy to improve the activities of daily living (ADL) and QOL of patients with chronic pain. Exercise is the most commonly recommended self-management strategy, and exercise therapy has been shown to be useful in improving pain and impairment in chronic pain patients^5^. However, the underlying mechanisms by which exercise therapy improves chronic pain are not fully understood.

Recent neuroimaging studies have suggested that chronic pain induces plastic reorganization of the nucleus in the brain and several neuronal pathways and circuits, suggesting that several critical regions to establish a chronic pain state^6,7^. The mesocorticolimbic system is an emotional/affective circuit in the brain that consists of the ventral tegmental area (VTA), nucleus accumbens (NAc), medial prefrontal cortex (mPFC), amygdala (Amyg), and hippocampus (HPC). There is abundant evidence showing that the mesocorticolimbic system plays an important role in regulating or amplifying chronic pain^8–10^, suggesting that dysfunction of the mesocorticolimbic system may be a cause of the chronification of pain.

Voluntary running (VR) or treadmill running in animal models of neuropathic pain (NPP) and inflammatory pain (IFP) are well known to markedly improve pain-related behaviors, such as mechanical allodynia and thermal hyperalgesia (exercise-induced analgesia: EIH)^11–13^. Kami et al. examined the relationship between the mesocorticolimbic system and EIH effects and indicated that VR activates dopamine (DA) neurons in the lateral-VTA, and as a result, produces EIH effects. Furthermore, they reported that suppression of GABAergic neurons in the central nuclei of amygdala (CeA) and activation of glutamate (Glu) neurons in the medial basolateral Amyg (medBA) projecting to the lateral shell of the NAc following VR may be a mechanism that produces EIH effects in NPP model mice. Additionally, many studies have suggested that multiple events, including significant changes in cytokines, neurotrophins, neurotransmitters, and endogenous opioids in the peripheral nerves, dorsal root ganglia, spinal cord dorsal horn, and brainstem, are possible mechanisms that induce EIH effects^14^.

The HPC, which is a component of the mesocorticolimbic system, is an important brain region that regulates cognition and emotions. The HPC is divided into the dorsal HPC (dHPC) and ventral HPC (vHPC) by genetic and functional characteristics in the long axis direction of the HPC^15^. The dHPC functions involve memory storage and spatial navigation^16,17^, while the vHPC is an important component of emotions, especially anxiety and depression^18–20^. In addition, pharmacological or optogenetic activation of the dHPC in NPP model rats improves pain-related behaviors^21^, and suppression of dHPC activity underlies cognitive deficits in chronic pain^22^. These results suggest that the dHPC is involved in pain perception and cognition. In contrast, the vHPC is more involved in the affective dimensions of pain^23^, and it has been suggested that depression and anxiety disorders with chronic pain are closely associated with the vHPC^24^. In addition, abnormal avoidance behaviors associated with depression and anxiety interact cyclically with pain. These findings suggest that the vHPC is an important brain region that modulates the chronification of pain in its affective dimensions.

At least 21 types of interneurons have been identified in the HPC-CA1, which support different neural activities and cognitive processing^25^. Parvalbumin (PV)-positive interneurons account for 24% of all GABAergic cells in the HPC-CA1 and have been shown to be involved in diverse brain functions, including pyramidal cell regulation, synaptic plasticity, and network oscillation generation^26–28^. Somatostatin (SOM)-positive interneurons, another important subtype of HPC inhibitory interneurons, comprise approximately 12% of all GABAergic cells in the HPC-CA1^29^, which are slower spiking interneurons^27^. Although both HPC PV^+^ and SOM^+^ interneurons inhibit excitation of pyramidal neurons, they may have distinct roles because of their different layer distribution and morphological and electrophysiological properties^25,27,30,31^.

Although the HPC may play a critical role in inducing the chronification of pain, the relationship between the vHPC, which is closely involved with emotions, and EIH effects has never been investigated. Therefore, we examined the effects of VR on FosB^+^ cells and GABAergic interneurons in the vHPC-CA1 of NPP model mice. The results of the present study indicated that suppression of the activated neurons via activation of GABAergic interneurons in the vHPC-CA1 by VR might provide a mechanism to produce EIH effects, and suggested that disappearance of negative emotions such as fear and anxiety due to exercise may play a critical role in improving chronic pain.

## Results

### Changes in running distances and pain behaviors

**Figure 1:**
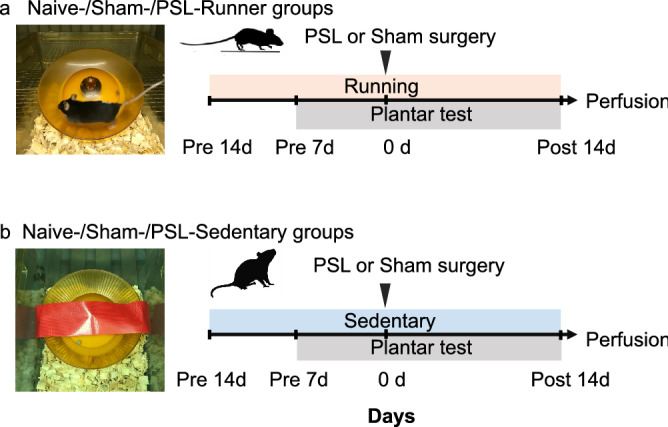
Protocol for voluntary running. (**a**) Naive-, Sham- and PSL-Runner mice were allowed to run freely on the running wheel, whereas (**b**) Naive-, Sham- and PSL-Sedentary mice were reared in cages with a locked running wheel. Mice in all experimental groups were placed in individual cages. *PSL* partial sciatic nerve ligation.

The effectiveness of forced exercise in producing EIH has been demonstrated in many studies^32,33^. Conversely, VR not only produces significant EIH effects in several animal models of pain, but also improves symptoms such as depression and anxiety-like behaviors^19,34,35^. Therefore, we used VR as an exercise method to elucidate the mechanism of EIH effects in the present study, and the VR protocol is shown in Fig. 1.

**Figure 2:**
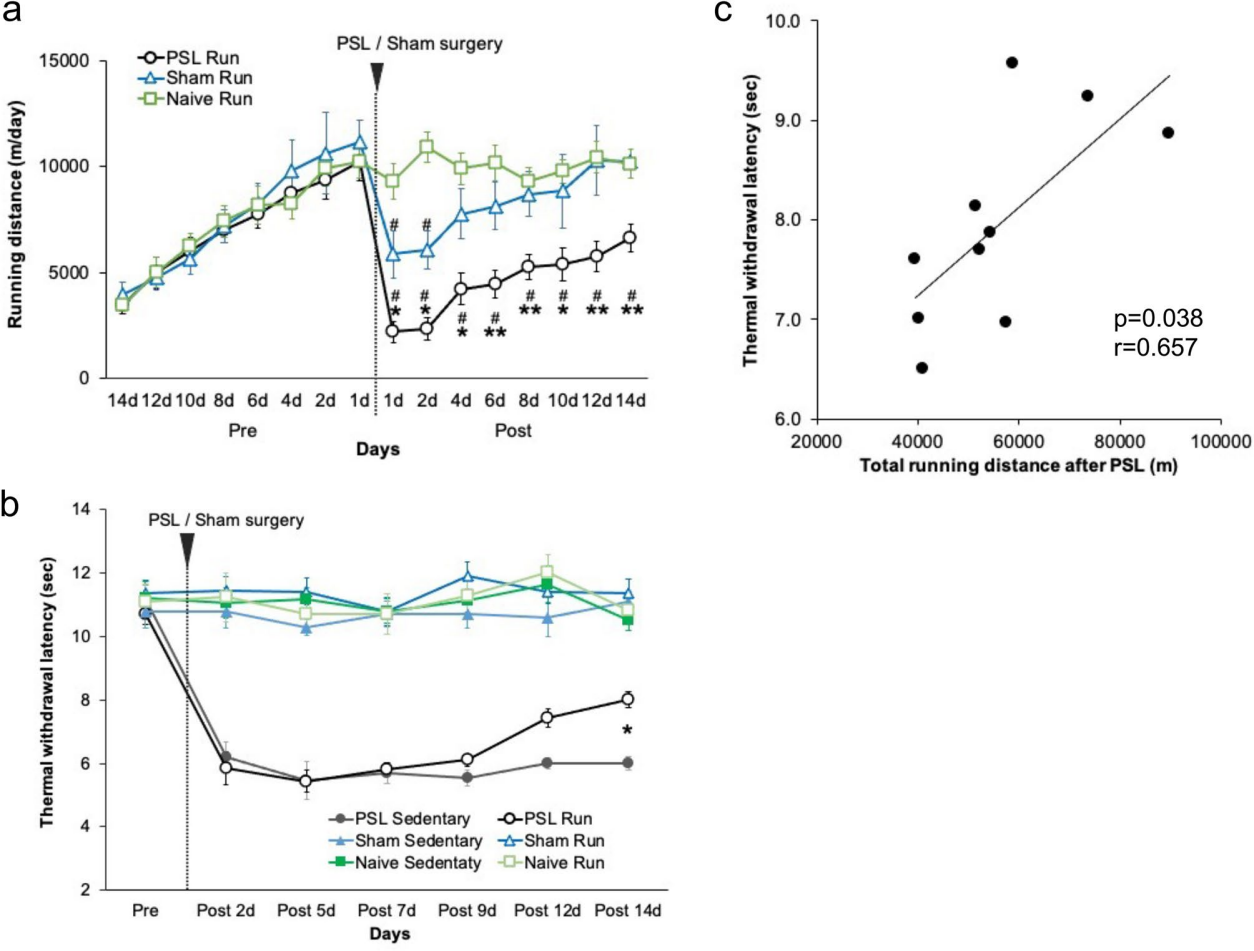
Changes in running distances and pain thresholds during the experimental period. (**a**) Changes in running distances in the three Runner groups before and after surgery (n = 9–10). Running distances of PSL-Runner mice gradually increased after PSL surgery. Data represent the mean ± SEM. A two-way repeated measures analysis of variance followed by Tukey’s post-hoc test was used to compare the Running distance among PSL-/Sham-/Naive-Runner groups. *p < 0.05, **p < 0.01, compared to Sham-Runner mice. ^#^p < 0.01, compared to Naive-Runner mice. (**b**) Thermal withdrawal latencies were significantly higher in PSL-Runner mice than in PSL-Sedentary mice at days 14 post-surgery. Data represent mean ± SEM. A three-way repeated measures analysis of variance followed by Tukey’s post-hoc test was used to compare the withdrawal latencies among all experimental groups. *p < 0.01, PSL-Runner mice compared to PSL-Sedentary mice (n = 9–10). (**c**) Relationship between thermal withdrawal latency and total running distance after PSL surgery in PSL-Runner mice. A significant positive correlation was observed between the two variables (p = 0.038, r = 0.657, n = 10). *PSL* partial sciatic nerve ligation, *SEM* standard error of the mean.

Figure 2a shows the changes in running distance per day in Naive, Sham, and partial sciatic nerve ligation (PSL)-Runner mice. The mean running distance in the three groups was approximately 3500 m/day on day 1 after the start of the experiment (Pre14 d), and running distances markedly increased by day 14 (Pre1 d) (Naive-Runner: 10,251 ± 818 m/day; Sham-Runner: 11,144 ± 1060 m/day; and PSL-Runner: 10,255 ± 906 m/day). The running distance in Sham-Runner mice decreased to 5867 ± 1100 m/day on day 1 post-surgery (1 day), but these values returned to near pre-surgery levels by day 14 post-surgery (10,217 ± 1600 m/day: 91.7%). In contrast, the running distance in PSL-Runner mice was markedly decreased on day 1 post-surgery (2199 ± 514 m/day: 21.4%). However, these values gradually increased thereafter, and the running distance in PSL-Runner mice recovered to approximately 64.9% (6656 ± 640 m/day) of the pre-surgery level by day 14 post-surgery (14 days). PSL-Runner mice indicated significantly lower daily running distance than the Naive-Runner and Sham-Runner mice from day 1 post-surgery (1 day) to day 14 post-surgery (14 day) (time effect, F_(15, 375)_ = 24.59, p < 0.001, surgery effect, F_(2,25)_ = 4.565, p = 0.02, interaction, F_(30, 375)_ = 7.684, p < 0.001, Two-way repeated measures ANOVA followed by Tukey’s post-hoc test).

We performed plantar tests to evaluate the effects of VR on heat hyperalgesia in PSL mice (Fig. 2b). A three-way repeated-measures ANOVA (surgery × exercise × time) followed by Tukey’s post hoc test (surgery effect , F_(2, 36)_ = 133.0, p < 0.001, exercise effect, F_(1,36)_ = 4.115, p = 0.049, time effect, F_(6, 216)_ = 16.25, p < 0.001, surgery × exercise, F_(2,36)_ = 0.839, p = 0.440, surgery × time, F_(12, 216)_ = 15.05, p < 0.001, exercise × time, F_(6,216)_ = 0.923, p = 0.479, surgery × exercise × time, F_(12,216)_ = 0.872, p = 0.575) indicates significant interaction surgery × time, and significant main effects of surgery, exercise and time. No marked differences were noted in the pre-surgery data of withdrawal latencies among the mice assigned to the six experimental groups. Furthermore, Runner mice in the Naive and Sham groups exhibited no significant alterations in withdrawal latencies throughout the experimental period, indicating that VR had no effect on sensory sensitivity in mice without PSL. PSL-Sedentary and PSL-Runner mice exhibited a markedly shorter withdrawal latency at 2 days after PSL (PSL-Sedentary: 6.2 ± 0.3 s, PSL-Runner: 5.8 ± 0.5 s) compared to pre-surgery levels (PSL-Sedentary: 11.2 ± 0.3, PSL-Runner: 10.7 ± 0.3 s). In PSL-Sedentary mice, decreased latencies were maintained throughout the experimental period. Conversely, withdrawal latencies in PSL-Runner mice were significantly increased on day 14 compared with those of PSL-Sedentary mice (PSL-Runner: 8.0 ± 0.3, PSL-Sedentary: 6.0 ± 0.2 s, p = 0.002). These results indicated that the pain behaviors in NPP model mice were significantly improved by the VR protocol.

Figure 2c shows the relationship between withdrawal latencies at 14 days post PSL and total running distances during the 14-day period after PSL in PSL-Runner mice. We found a significant positive correlation between total running distance and withdrawal latency (Fig. 2c; p = 0.038, r = 0.657, n = 10).

### VR inhibits PSL-induced FosB^+^ cells in the vHPC-CA1

**Figure 3:**
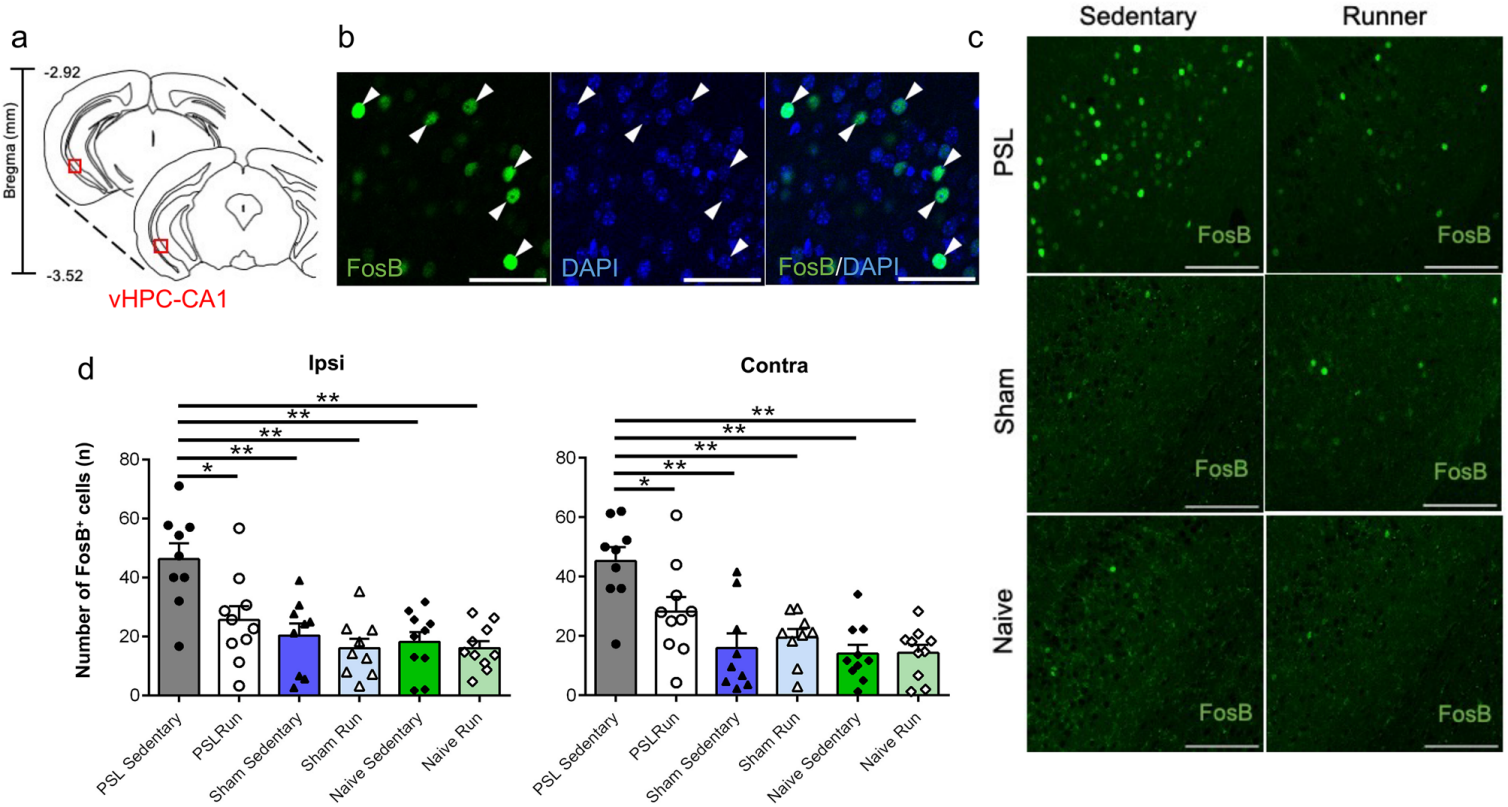
Effects of PSL and voluntary running on FosB^+^ cells in the vHPC-CA1. (**a**) Representative coronal brain images including the vHPC-CA1. The vHPC-CA1 is highlighted by the red squares. (**b**) Photomicrograph showing FosB^+^ (green)/DAPI (blue) immunoreactivities in the vHPC-CA1. The arrowheads indicate activated cells marked by double staining with FosB antibody and DAPI. Scale bar = 50 μm. (**c**) Photomicrographs showing FosB (green) immunoreactivity in the vHPC-CA1. Many FosB^+^ cells in the vHPC-CA1 were detected in PSL-Sedentary mice compared to other experimental groups. Scale bars = 100 μm. (**d**) Bar charts showing the number of FosB^+^ cells in the vHPC-CA1 in all experimental groups. The number of FosB^+^ cells in the vHPC-CA1 in PSL-Sedentary mice was significantly increased on both the ipsilateral and contralateral sides of the PSL surgery. Bars represent mean ± SEM. A two-way analysis of variance followed by Tukey’s post hoc-test was used to compare the number of FosB^+^ cells among the experimental groups. *p < 0.01, **p < 0.001. n = 9–10. *PSL* partial sciatic nerve ligation, *SEM* standard error of the mean, *vHPC* ventral hippocampus.

Previous studies demonstrated that the HPC-CA1 can undergo remarkable changes with pain and exercise^36,37^. Thus, the HPC-CA1 are an interesting region to execute exercise and pain researches. In addition, neurons located in the CA1 are known to project to the Amyg, a pathway associated with fear^38^. Based on these findings, we considered that the CA1 may be a critical brain region to produce EIH effect. There is an animal study used only FosB immunostaining to know effects of VR on the HPC^39^, which suggested that single immunostaining with FosB antibody is a worthy effort to know activation or inactivation of neurons located in the vHPC-CA1 in response to PSL and VR. Therefore, in this study, we marked activated cells by immunostaining with FosB antibody and DAPI in the CA1 (Fig. 3a,b), and they were counted as FosB^+^ cells. It has been known that nuclei expressing FosB protein represent activated cells, because FosB protein within the nuclei gradually accumulates in response to chronic stimuli, including wheel running^39^, and its accumulation persists for long periods of time because of its high stability^40^.

Figure 3c shows typical images of FosB immunostaining in vHPC-CA1 in all experimental groups. FosB^+^ cells were abundantly and homogeneously expressed in the stratum pyramidale of the vHPC-CA1. Next, we compared the number of FosB^+^ cells in the vHPC-CA1 in the ipsilateral and contralateral sides among each group (Fig. 3d). The number of FosB^+^ cells was significantly higher in PSL-Sedentary mice (Ipsi: 46.2 ± 5.4, Contra: 45.2 ± 4.7) than in PSL-Runner mice (Ipsi: 25.5 ± 4.7, Contra: 28.7 ± 4.8), Sham-Sedentary mice (Ipsi: 20.3 ± 4.2, Contra: 15.9 ± 5.0), Sham-Runner mice (Ipsi: 16.0 ± 3.3, Contra: 19.5 ± 2.9), Naive-Sedentary mice (Ipsi: 18.2 ± 3.3, Contra: 14.0 ± 3.0), and Naive-Runner mice (Ipsi: 16.1 ± 2.4, Contra: 14.3 ± 2.7) (Ipsi: exercise effect, F_(1, 51)_ = 7.687, p = 0.008, surgery effect, F_(2,51)_ = 14.07, p < 0.001, interaction, F_(2, 51)_ = 3.291, p = 0.045. Contra: exercise effect, F_(1, 51)_ = 1.790, p = 0.187, surgery effect, F_(2,51)_ = 18.78, p < 0.001, interaction, F_(2, 51)_ = 3.817, p = 0.029. Two-way ANOVA followed by Tukey’s post-hoc test). No difference in the number of FosB^+^ cells was observed between the ipsilateral and contralateral sides (Fig. 3d).

**Figure 4:**
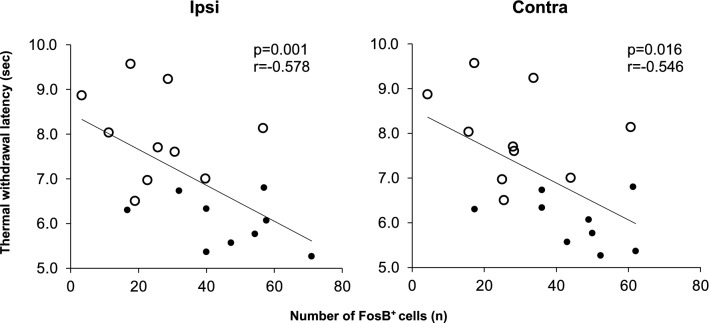
Relationship between the number of FosB^+^ cells in the vHPC-CA1 and thermal withdrawal latencies in PSL-mice. A significant negative correlation was observed between the number of activated (FosB^+^) neurons in the vHPC-CA1 and thermal withdrawal latencies in PSL-Sedentary mice (black circles) and PSL-Runner mice (white circles). Ipsilateral side: p = 0.001, r = 0.578, n = 19, Contralateral side: p = 0.0016, r = 0.546. n = 19. *PSL* partial sciatic nerve ligation, *vHPC* ventral hippocampus.

When data from both PSL-Runner and PSL-Sedentary mice were integrated into the analysis, significant negative correlations between the number of FosB^+^ cells in the vHPC-CA1 and thermal withdrawal latencies were detected for both the ipsilateral (p = 0.009, r = − 0.578) and contralateral sides (p = 0.016, r = − 0.546) (Fig. 4), suggesting that activation of neurons in the vHPC-CA1 may determine the levels of heat hyperalgesia after PSL.

### VR induces activation of GABAergic interneurons in the vHPC-CA1

GABAergic interneurons are important local elements in the brain circuitry and regulate the excitability of pyramidal neurons in the vHPC-CA1^41–43^. As estimated, 17.5% of GABAergic neurons in the vHPC-CA1 are classified as PV^+^ cells, with the same proportion classified as SOM^+^ cells as well^44^.

**Figure 5:**
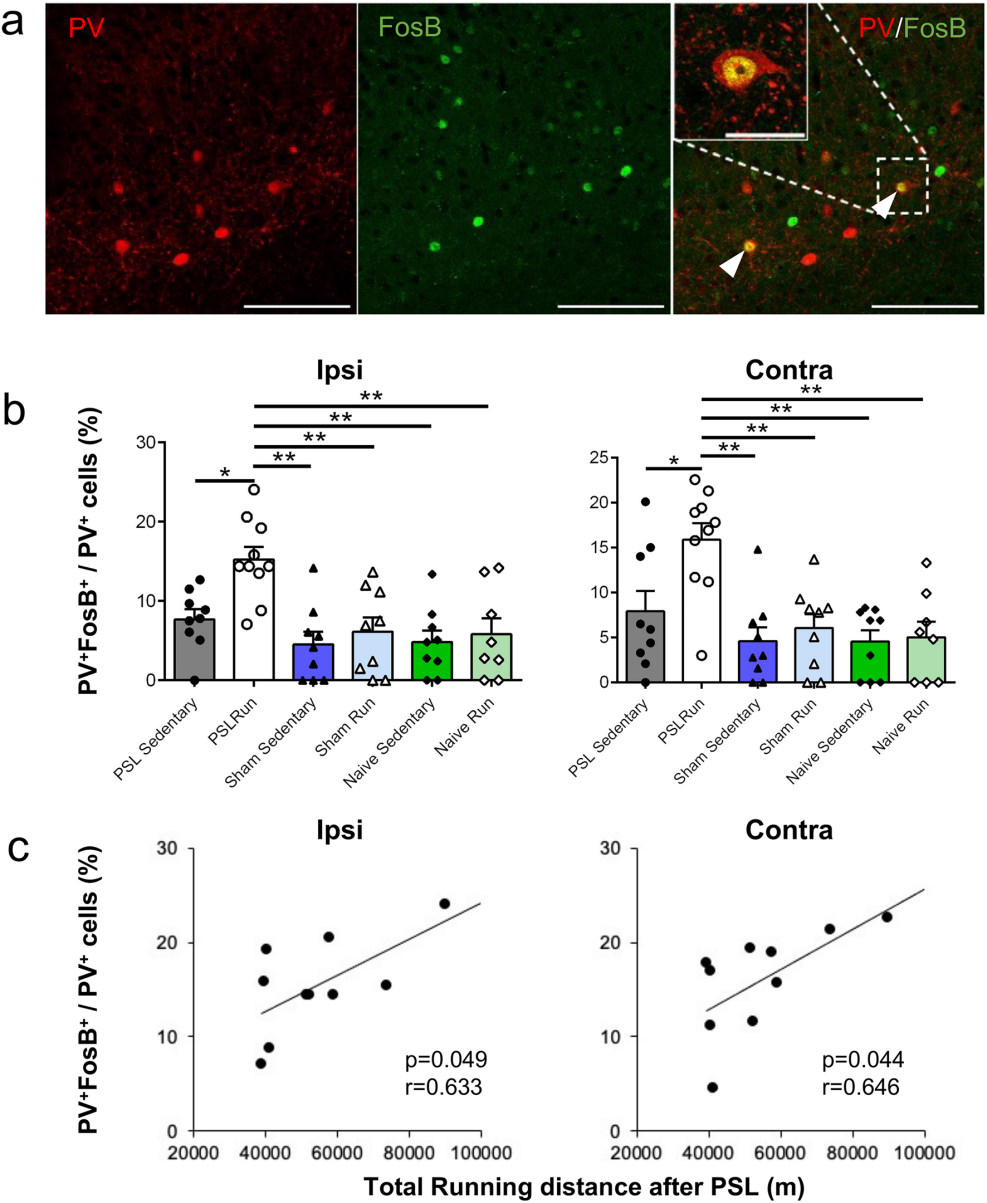
Voluntary running induces activation of PV^+^ interneurons in the vHPC-CA1. (**a**) Representative photomicrographs showing PV (red) and FosB (green) immunoreactivities in the vHPC-CA1 in PSL-Runner mice. Many PV^+^ FosB^+^ neurons (arrowheads) were detected in PSL-Runner mice. Scale bar = 100 mm. An enlarged PV^+^ FosB^+^ neuron is shown in the left upper corner. Scale bar = 25 mm. (**b**) Bar charts showing the proportion of PV^+^ FosB^+^ cells in the vHPC-CA1. The proportion of PV^+^ FosB^+^ cells in PSL-Runner mice is significantly higher than in other groups. Bars represent mean ± SEM. A two-way analysis of variance followed by Tukey’s post hoc-test was used to compare the proportion of PV^+^ FosB^+^ cells among the experimental groups. *p < 0.01, **p < 0.001. (**c**) Relationship between the proportion of PV^+^ FosB^+^ cells and the total running distance after PSL surgery in PSL-Runner mice. Significant positive correlations were observed in both the ipsilateral (p = 0.049, r = 0.633, n = 10) and the contralateral sides (p = 0.044, r = 0.646, n = 10). *PSL* partial sciatic nerve ligation, *vHPC* ventral hippocampus.

When brain sections, including the vHPC-CA1, were double-immunostained with PV and FosB antibodies, most of the PV immunoreactive neurons that were observed as fusiform were widely scattered in the stratum pyramidale and the stratum oriens in the vHPC-CA1, and strong PV^+^ immunoreactivity was detected in the cell bodies of neurons (Fig. 5a). Such characteristics of PV^+^ cells are consistent with those found in a previous study^45^.

Figure 5b shows the proportion of PV- and FosB-double-positive cells (activated PV^+^ interneurons) in the vHPC-CA1 in all experimental groups. The proportion of activated PV^+^ interneurons (PV^+^ FosB^+^/PV^+^) in PSL-Runner mice was significantly higher (Ipsi: 14.7 ± 1.3%, Contra: 15.7 ± 1.8%) than in PSL-Sedentary mice (Ipsi: 7.7 ± 1.3%, Contra: 7.9 ± 2.3%), Sham-Sedentary mice (Ipsi: 4.5 ± 1.6%, Contra: 4.6 ± 1.6%), Sham-Runner mice (Ipsi: 6.1 ± 1.8%, Contra: 6.1 ± 1.5%), Naive-Sedentary mice (Ipsi: 4.8 ± 1.4%, Contra: 4.6 ± 1.3%), and Naive-Runner mice (Ipsi: 5.8 ± 2.0%, Contra: 5.0 ± 1.8%), suggesting that the activation of PV^+^ interneurons may be enhanced via a specific signal(s) that is produced by concurrent stimulation of both PSL and VR (Ipsi: exercise effect, F_(1, 48)_ = 6.445, p = 0.014, surgery effect, F_(2,48)_ = 9.802, p < 0.001, interaction, F_(2, 48)_ = 2.546, p = 0.089. Contra: exercise effect, F_(1, 48)_ = 5.346, p = 0.025, surgery effect, F_(2,48)_ = 10.54, p < 0.001, interaction, F_(2, 48)_ = 2.763, p = 0.073. Two-way ANOVA followed by Tukey’s post-hoc test). Remarkable differences were not detected in the proportion of activated PV^+^ interneurons between the ipsilateral and contralateral sides.

We found significant positive correlations between the proportion of activated PV^+^ cells and total running distance after PSL surgery in PSL-Runner mice on both the ipsilateral (p = 0.0497, r = 0.6325) and contralateral sides (p = 0.0436, r = 0.6461) (Fig. 5c). These results suggest that the amount of VR after PSL may be an important factor that affects the degree of activation of the PV^+^ neurons in the vHPC-CA1.

**Figure 6:**
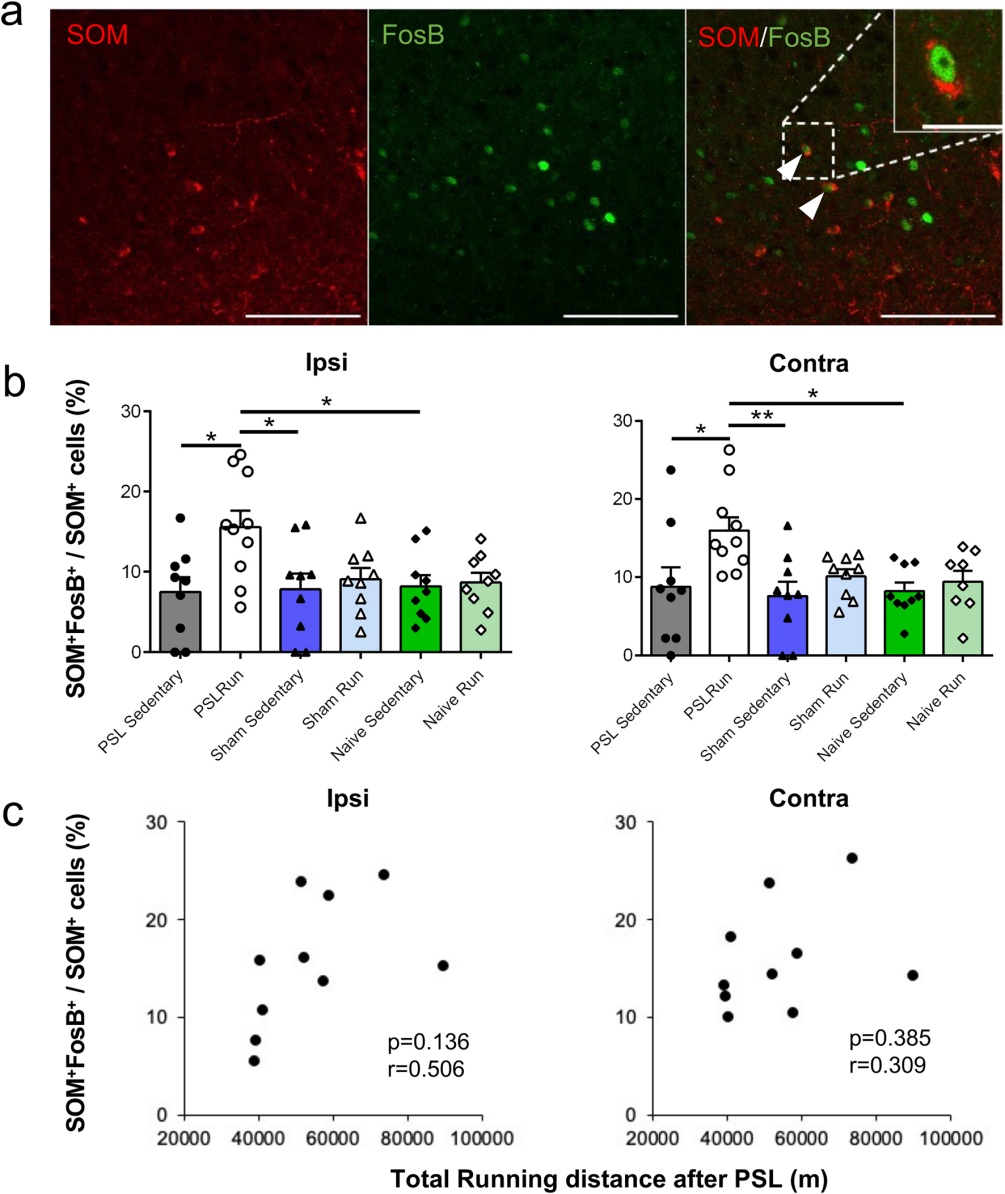
Voluntary running induces activation of SOM^+^ interneurons in the vHPC-CA1. (**a**) Representative photomicrographs showing SOM (red) and FosB (green) immunoreactivities in the vHPC-CA1 in PSL-Runner mice. Many SOM^+^ FosB^+^ neurons (arrowheads) were detected in PSL-Runner mice. Scale bar = 100 mm. An enlarged SOM^+^ FosB^+^ neuron is shown in the left upper corner. Scale bar = 25 mm. (**b**) Bar charts showing the proportion of SOM^+^ FosB^+^ cells in the vHPC-CA1. The proportion of SOM^+^ FosB^+^ cells in PSL-Runner mice was significantly higher than other groups. Bars represent mean ± SEM. A two-way analysis of variance followed by Tukey’s post hoc-test was used to compare the proportion of PV^+^ FosB^+^ cells among the experimental groups. *p < 0.05, **p < 0.01. (**c**) Relationship between the proportion of SOM^+^ FosB^+^ cells and total running distance after PSL surgery in PSL-Runner mice. No significant correlation was observed on the ipsilateral side (p = 0.136, r = 0.506, n = 10) or the contralateral side (p = 0.385, r = 0.309, n = 10). *PSL* partial sciatic nerve ligation, *SEM* standard error of the mean, *vHPC* ventral hippocampus.

Brain sections, including the vHPC-CA1, were double-immunostained with SOM and FosB antibodies. Figure 6a shows that in the vHPC-CA1, the majority of SOM immunoreactive neurons were located in the stratum oriens and that the immunoreactivities were concentrated in the perinuclear regions (Fig. 6a). These results are consistent with previous observations^45^.

Among the experimental groups, only the proportion of activated SOM^+^ interneurons (SOM^+^ FosB^+^/SOM^+^) in PSL-Runner mice were significantly higher (Ipsi: 15.6 ± 2.1, Contra: 15.9 ± 1.7) than in PSL-Sedentary mice (Ipsi: 7.5 ± 1.9%, Contra: 8.8 ± 2.5%), Sham-Sedentary mice (Ipsi: 7.8 ± 2.0%, Contra: 7.6 ± 1.8%), and Naive-Sedentary mice (Ipsi: 8.2 ± 1.4%, Contra: 8.2 ± 1.0%) (Fig. 6b). However, no statistically significant difference was found among the Runner groups (data not shown), suggesting that VR may induce activation of SOM^+^ interneurons in the vHPC-CA1 (Ipsi: exercise effect, F_(1, 49)_ = 5.578, p = 0.022, surgery effect, F_(2, 49)_ = 2.244, p = 0.117, interaction, F_(2, 49)_ = 3.071, p = 0.055. Contra: exercise effect, F_(1, 48)_ = 7.028, p = 0.011, surgery effect, F_(2,48)_ = 3.032, p = 0.058, interaction, F_(2, 48)_ = 1.815, p = 0.174. Two-way ANOVA followed by Tukey’s post-hoc test). Similarly, in the case of PV^+^ interneurons, a combination of PSL and VR may induce further activation of SOM^+^ interneurons. The proportion of activated SOM^+^ interneurons between the ipsilateral and contralateral sides did not show significant differences among the six experimental groups (Fig. 6b).

Unlike in the case of activated PV^+^ interneurons, we did not detect a significant correlation between the percentage of activated SOM^+^ interneurons and the total running distance after PSL in PSL-Runner mice (Fig. 6c) (ipsilateral side: p = 0.136, r = 0.506; contralateral side: p = 0.385, r = 0.309).

### Activation of neurons projecting from the vHPC-CA1 to the basolateral amygdala

**Figure 7:**
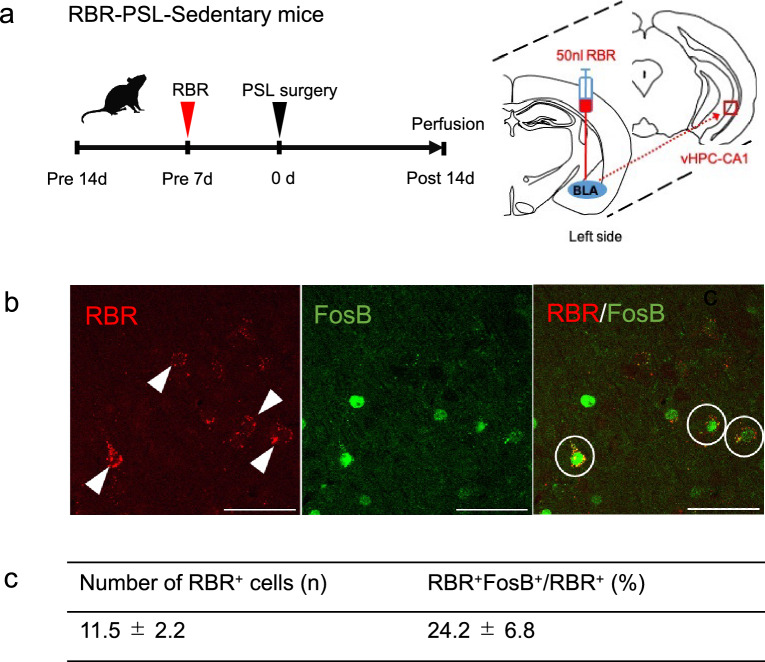
Activation of neurons projecting from the vHPC-CA1 to the BLA in PSL-sedentary mice. (**a**) Protocol for the retrograde tracer experiment. PSL-sedentary mice were injected with 50 nl of RBR into the left side of the BLA one week before PSL surgery. At 2 weeks later PSL surgery, mice were perfused and analyzed with double-immunostaining (n = 5). (**b**) Photomicrographs showing RBR (red) positive signals and FosB (green) immunoreactivities in the vHPC-CA1. Arrowheads indicate neurons (RBR^+^ cells) projecting from the vHPC to the BLA (left panel). Activated neurons (RBR^+^ FosB^+^ cells) projecting from the vHPC to the BLA are marked by the circles in the right panel. Scale bars = 50 μm. (**c**) A table showing the quantitative results of activated neurons (RBR^+^ FosB^+^ cells) projecting from the vHPC to the BLA in PSL-Sedentary mice. Quantitative data are presented as mean ± SEM. *PSL* partial sciatic nerve ligation, *vHPC* ventral hippocampus, *BLA* basolateral amygdala, *RBR* Retrobeads Red.

It has been known that the basolateral amygdala (BLA) is an important brain region in regulating pain-related emotions and fear memories, and our previous study indicated that NPP induces remarkable activation of GABAergic neurons located in the BLA^46^. Therefore, to know whether activation of projection neurons in the CA1 of NPP model mice is a cause to activate BLA-GABAergic neurons, we performed the Retrobeads Red (RBR) experiment on NPP model mice (Fig. 7a). We did not perform the RBR experiment in PSL-Runner mice, because VR significantly reduced the FosB immunoreactivities. We used a combination of retrograde tracing and FosB immunostaining to investigate whether neurons projecting from the vHPC-CA1 to the BLA were activated by PSL surgery. The brains of five mice, in which RBRs were injected precisely into the BLA, were used for immunostaining.

We analyzed the vHPC-CA1 on the ipsilateral side of the injection because RBR-incorporated neurons were detected only on the left side of the vHPC-CA1. RBR^+^ neurons were observed within the set square of the vHPC-CA1 (11.5 ± 2.2, n = 5), and 24.2 ± 6.8% of these RBR^+^ neurons showed FosB^+^ immunoreactivity (Fig. 7b,c). These results indicate that NPP can activate neurons that project from the vHPC- CA1 to the BLA.

## Discussion

The present study indicated that VR for 2 weeks before and after PSL surgery significantly improved pain behaviors on Post 14 days. Previous studies reported more striking effects of VR in the plantar test^23,46^. There are some reasons producing the differences of pain thresholds between the previous and the present study. (1) The difference in measurement time (8:00 AM in the previous studies and 18:00 in this study). Actually, a previous study reported that NPP model animals exhibit diurnal variations in pain intensity^47^. (2) Individual differences among mice may also influence to the results, and in fact, the running distances were slightly less than those of the previous studies. Furthermore, (3) the examiners were different in the previous and the present study.

We found positive correlations between thermal withdrawal latencies and total running distances after PSL surgery, suggesting that more active VR can enhance the EIH effect. Many studies have demonstrated that whole-body exercise can induce analgesic effects in patients with chronic pain and pain model animals^11–13,23,32,33,46^. However, the characteristics of exercise such as the intensity, amount, duration, and type that produce sufficient EIH effects have not been sufficiently investigated, and it is also unknown whether differences in sex, diseases, and pain models are involved in the expression of EIH effects. Therefore, further efforts to elucidate these issues are necessary to advance EIH research.

We found that more intense immunoreactivity of FosB was detected in larger nuclei in the CA1 by double staining with FosB antibody and DAPI. These FosB^+^ cells may be neurons, but not glial cells, because it has been known that nuclear size of neurons is larger compared to glial cells^48,49^. It has been reported that neurons in the vHPC-CA1 are activated in mice exposed to anxiety-induced environments, and that the majority of these neuronal populations are Glu neurons, suggesting that activation of Glu neurons promotes avoidance behavior^19^. Glu neurons projecting to the NAc in the vHPC are activated in mice exhibiting depression-like behavior due to chronic social defeat stress^50^. In the HPC-CA1, there are 91% of Glu neurons and 9% of GABAergic neurons in the dHPC, and 82% of Glu neurons and 18% of GABAergic neurons in the vHPC^44^. Therefore, based on the reports and the result of our double staining with FosB and DAPI, most of FosB^+^ cells localized in the HPC-CA1 may correspond to activated Glu neurons, although accurate neuronal population of FosB^+^ cells in the CA1 in chronic pain state should be determined by using latest technologies.

Sciatic nerve chronic constriction injury (CCI) model rats showing behavioral deficits in the radial maze test showed an increase in FosB/ΔFosB expression in neurons located in the vHPC^51^. These observations were consistent with the present results, in which PSL-Sedentary mice showed an increased number of FosB^+^ cells in the vHPC-CA1. These changes suggest that NPP induces not only abnormalities in the sensory-discriminative component, but also hyperexcitation of neurons in the vHPC-CA1, and may result in deterioration of the emotional and affective aspects associated with chronic pain. Chronic pain is often accompanied by psychiatric disorders such as anxiety and depression^24^. Emotions such as fear and anxiety associated with pain are known to cause a vicious cycle of further aggravation and intractable pain through excessive defensive responses and avoidance behaviors^52^, suggesting that these may contribute to persistent pain in PSL-Sedentary mice.

In this study, we focused on changes in neurons in the vHPC, but not in the dHPC, because they play an important role in the emotional changes associated with chronic pain. Reportedly, neural connectivity of the vHPC-CA1 and mPFC pathways is associated with pain behavior in mouse models of peripheral inflammatory pain, and improved neural connectivity of this pathway improves pain behaviors^36^. Furthermore, increased neurogenesis of the vHPC dentate gyrus attenuates pain behaviors^23^. IL-1β is increased along with FosB in the vHPC of CCI mice^51^, and an increase in hippocampal IL-1β correlates with tactile allodynia^53^, indicating that the vHPC as with the dHPC receives noxious stimuli, thereby generating multiple responses as mentioned above. In the present study, we found that VR can decrease the number of FosB^+^ cells in the vHPC-CA1, and that thermal hyperalgesia was significantly improved in PSL-Runner mice. Furthermore, the number of FosB^+^ neurons in the vHPC-CA1 negatively correlated with the degree of thermal hyperalgesia. These results suggest that the suppression of activated neurons in the vHPC-CA1 by VR may be a mechanism to produce EIH effect. Since activation of neurons in the vHPC-CA1 is associated with the appearance of negative emotions such as fear and anxiety, it has been suggested that disappearance of negative emotions via suppression of these neurons by VR may play an important role in the production of EIH.

A previous study reported that VR increased FosB/ΔFosB expression in the vHPC of mice^39^. However, we found that the number of FosB^+^ cells are not increased by VR. These are some possible causes producing this difference. In the present study, mice were kept individually in cages with running wheels, whereas Nishijima et al.^39^ are kept in groups of five mice. Furthermore, they reported a positive correlation between neurogenesis and the number of FosB^+^ cells. It has been known that hippocampal neurogenesis by VR is suppressed in rats which were housed individually, while rats which were housed in groups increase hippocampal neurogenesis by VR^54^. Thus, the difference of social environment may be responsible for the difference in FosB^+^ cell counts. In addition, the differences in FosB antibody and the image analysis to detect immune responses used in two studies also may be involved to the results of FosB counting.

Inhibitory interneurons, PV^+^, and SOM^+^ neurons differ in morphology, electrophysiological properties, and binding specificity, suggesting that they assume different functions^25,30,31^. The present results show that the number of activated PV^+^ and SOM^+^ neurons was increased in PSL-Runner mice in the vHPC-CA1. It has been shown that 4 weeks of VR increases GAD67 mRNA expression in all hippocampal subfields and enhances GABA synthesis capacity in GABA interneurons^55^. Moreover, VR increases extracellular GABA levels and the number of activated GABAergic interneurons in the vHPC and suppresses stress-induced pyramidal cell activation^35^. In accordance with these previous findings, we found that VR also induced the activation of PV^+^ and SOM^+^ interneurons in the vHPC-CA1 of the NPP model, suggesting that VR can reinforce the inhibitory effects on neurons located in the vHPC-CA1.

In the present study, the increased levels of activated PV^+^ and SOM^+^ interneurons were higher than those in Sham- and Naive-Runner mice. It has been known that extracellular GABA levels in the vHPC of Runner mice were increased by cold water stress, but this response was not induced in Sedentary mice^35^. Likewise, we found that activation of PV^+^ and SOM^+^ interneurons in the vHPC-CA1 was remarkably enhanced by combination of both PSL and VR. Thus, although it suggests that concurrent stimulation of both PSL and VR acts synergistically on PV^+^ and SOM^+^ interneurons, possible mechanism to produce this effect is unknown. A significant positive correlation between the number of activated PV^+^ interneurons, but not SOM^+^ interneurons, and running distances after surgery in PSL-Runner mice was also found in this study. Arriaga and Han^37^ reported a significant positive correlation between running speed and neural activity of PV^+^ and SOM^+^ interneurons in the HPC-CA1. Furthermore, they indicated that the percentage of PV^+^ interneurons activating with exercise is greater than that of SOM^+^ interneurons. In addition, it has been shown that GAD67, which has been suggested to be increased in VR, is strongly expressed in PV^+^ interneurons among a subgroup of GABAergic neurons in the HPC-CA1 of mice^56^. These findings suggest that PV^+^ interneurons are more likely to be activated by running compared to SOM^+^ interneurons.

The present study also indicated that the neurons in the vHPC-CA1 react bilaterally to the same extent on PSL and VR stimuli. Interestingly, the increased expression of translocator protein (TSPO) in spared nerve injury (SNI) model rats has been detected in neurons and microglia in both ipsilateral and contralateral sides of the vHPC^57^. These findings suggest that the vHPC can receive bilaterally nerve injury and running stimuli, although exact pathways remain to be determined. In addition to PV^+^ and SOM^+^ interneuron, there are other subtypes of GABAergic interneurons which are marked by NPY and calretinin in the vHPC-CA1. Therefore, it cannot ignore a possibility that GABAergic interneurons other than PV^+^ and SOM^+^ interneuron may play critical roles in the vHPC-CA1 to produce EIH effects. Taken together, a potential target of GABAergic interneurons in the vHPC may be projecting neurons to Amyg, and GABAergic interneurons activated by VR may suppress the activation of principal neurons via a feedforward mechanism, and thereby may produce EIH effects.

NPP model mice show increased anxiety-like behaviors compared to Sham mice^58^. Pharmacological inactivation of the vHPC inhibits the expression of conditioning fear^59^, and activation of neurons projecting from the vHPC to the BLA acts on fear renewal^60^. In this study, our retrograde tracer experiment indicated that neurons projecting from the vHPC-CA1 to the BLA were activated in PSL-sedentary mice. Therefore, NPP activates neurons projecting from the vHPC-CA1 to the BLA, which may reinforce fear memory associated with chronicity of pain.

Exercise therapy is not only a treatment to improve ADL and QOL in patients with chronic pain, but also can improve chronic pain itself. In the present study, we showed that VR suppresses the activation of the FosB^+^ cells in the vHPC-CA1, which may be induced via feedforward inhibition by GABAergic interneurons, and thereby may contribute to the improvement of chronic pain. Furthermore, since activation of neurons in the vHPC-CA1 is associated with several negative emotions, our results suggest that the disappearance of fear and anxiety associated with chronic pain by VR may play a critical role in producing EIH effects. Thus, our findings further extend the effectiveness of exercise therapy to treat chronic pain from the point of view of psychological factors. Approaches to investigating the direct relationship between the EIH effect and fear memory will allow us to further elucidate the mechanisms of EIH effects.

## Methods

### Preparation of NPP model mice

Adult C57BL/6J mice were used in this study. NPP model mice were prepared by PSL^61^. Under deep anesthesia with 3% anesthetic isoflurane administered continuously through a mask at a flow rate of 1 L/min, approximately one-third to one-half of the sciatic nerve at the right mid-thigh level was tightly ligated using 8-0 silk sutures. Sham surgery was performed using the same procedures described above, excluding PSL. After surgery, the mice were monitored using individual cages and were returned to their usual cages after awakening. No any post-wound treatment was administered to the mice after surgery. However, several symptoms, such as the reduction of movement and body weight, swelling, excessive inflammation, and suppuration, were never observed in mice. All experiments were approved by the Animal Care Committee of Wakayama Medical University (Permit No. 977). All experiments conformed to the National Institutes of Health Guide for the Care and Use of Laboratory Animals 8th edition. This study was conducted in accordance with the Animal Research: Reporting on In Vivo Experiments (ARRIVE) guidelines.

### Experimental groups and the VR protocol

Mice were divided into six groups: (1) Naive-Sedentary mice (n = 9), (2) Naive-Runner mice (n = 9), (3) Sham-Sedentary mice (n = 9), (4) Sham-Runner mice (n = 9), (5) PSL-Sedentary mice (n = 9), and (6) PSL-Runner mice (n = 10). All Runner and Sedentary mice were placed in individual cages equipped with a running wheel or locked running wheel under a 12/12-h light–dark cycle (light on at AM 8:00), room temperature 23 ± 1 ℃, and were provided food and water ad libitum, and Naive, Sham, and PSL-Runner mice were allowed to run freely on the running wheel. Since running exercise in the early stage after nerve injury is an important requirement to induce significant EIH effects, the mice were returned to a cage equipped with a running wheel so that they could voluntarily run immediately after PSL surgery. All mice, except for Naive mice, underwent VR for 2 weeks prior to Sham or PSL surgery. PSL and Sham mice were returned to individual cages immediately after surgery, and running distances were measured during the 14-day period before and after surgery. The rotations from running on the running wheel/hour were monitored using a magnetic reed switch attached to a computerized exercise-monitoring system (SOF-860 wheel manager software, MED Associates, Inc.), and the daily running distances (m/day) were calculated using the number of rotations on the running wheel.

### Evaluation of pain behavior

To abolish diurnal variations in pain behavior, plantar tests were performed at 18:00. Prior to the plantar test, mice were placed in an acrylic glass enclosure (8.3 × 8.3 × 8.0 cm) and were allowed to acclimate for 30 min. Heat hyperalgesia was evaluated using the plantar test, which measures withdrawal latency (sec) to a radiant thermal stimulus delivered from beneath the glass floor to the plantar surface of the hind paw (Plantar Test, Model37570-001, Ugo Basile, Italy). The heat stimulus from the projector lamp bulb was focused on the plantar surface of the right hind paw, and the minimum time (sec) needed to evoke quick withdrawal of the paw due to heat stimulation was taken as the withdrawal latency. The plantar test was performed thrice for each mouse, and the mean values were considered as the withdrawal latency. In all behavioral evaluations, the investigator was blinded to the experimental groups to avoid bias.

### Immunofluorescence analysis

The primary antibodies used were as follows: FosB (mouse monoclonal antibody, dilution 1:2000, Abcam, ab11959, a marker of activated neurons), parvalbumin (polyclonal guinea pig antibody, dilution 1:1000, Synaptic System, Cat. No. 195 004, a marker of a specific population of GABAergic interneurons), Somatostatin-28 (polyclonal guinea pig antibody, dilution 1:500, Synaptic System, Cat. No. 366 004, a marker of a specific population of GABAergic interneurons).

Mice were perfused transcardially with 4% paraformaldehyde in 0.1 M phosphate-buffered saline (PBS), and the brains were post-fixed. After cryoprotection, the brains were frozen in dry, ice-cooled hexane. Serial 25-μm thick brain sections including the vHPC (Bregma − 3.5 mm to − 2.9 mm) in the coronal plane were mounted serially on slides. After blocking for non-specific staining, sections for dual immunofluorescence staining were incubated simultaneously with two types of primary antibodies diluted in 0.1 M PBS containing 5% normal donkey serum and 0.3% Triton X-100 at 4 ℃ for 48 h. Then, sections were incubated with the secondary antibodies diluted in 0.1 M PBS containing 5% normal donkey serum and 0.1% Triton X-100 during overnight at 4 ℃. The secondary antibody used for the detection of FosB immunoreactivity was Alexa Fluor 488-labeled donkey anti-mouse antibody (1:500, Abcam, ab150109). The secondary antibody used for the detection of PV and SOM immunoreactivity was Alexa Fluor 594-labeled donkey anti-guinea pig immunoglobulin-G (1:500, Jackson Immuno Research, 706-585-148). Sections were washed in 0.1 M PBS and mounted in Vectashield mounting medium with DAPI (H-1200, Vector Labs, Burlingame, CA, USA). Fluorescence signals were detected using a confocal microscope (LSM700, Carl Zeiss, Oberkochen, Germany) equipped with an argon-helium laser. Negative control sections processed without primary antibodies showed no significant positive immunoreactivity.

### Quantitative analysis of immunopositive neurons

Immunofluorescence images of the vHPC on the ipsilateral and contralateral sides were acquired at × 20 magnification. A square of 200 μm × 200 μm in size, as shown in Fig. 3a for the vHPC-CA1, was placed on the microscope images using ImageJ software (version 2.3.0/1.53q), and the number of immunopositive cells within the square was counted. The mean value of three randomly selected sections from the six sections per brain was regarded as the value of immunopositive neurons per mouse. To avoid any bias in the results of the quantitative analysis, the investigators were blinded to all experimental groups throughout the quantitative analyses.

### Injection of a retrograde tracer into the BLA, immunofluorescence staining, and quantitative analysis

Mice were placed in individual cages equipped with locked running wheel for 1 week prior to injection of the retrograde tracer and were then placed in a stereotaxic apparatus. Unilateral injection of Retrobeads Red (RBR) (50 nl, Lumafluor Inc., Naples, FL, USA) was performed by inserting a microsyringe into the left side of the BLA (Bregma: anteroposterior, − 1.46 mm; mediolateral, 2.9 mm; and dorsoventral, − 4.6 mm). RBR-injected mice were returned to their individual cages, and after 1 week, PSL surgery was carried out, and then the PSL-mice were kept during 2 weeks. The brains of RBR-injected Sedentary mice were processed in the same manner as that described for immunofluorescence analysis. To evaluate RBR-injection sites, 35-μm thick brain sections, including the BLA, were mounted serially, and after briefly rinsing with PBS, slides were mounted in Vectashield mounting medium with DAPI.

The same immunostaining procedure for FosB in the vHPC-CA1 was adapted for RBR-injected brain sections. Immunofluorescence images of the vHPC-CA1 on the administered sides of the RBR were acquired at a magnification of × 20. A square of 200 mm × 200 mm in size, as shown in Fig. 7 for the vHPC-CA1, was placed on the microscope images using ImageJ software (version 2.3.0/1.53q), and the number of RBR-positive cells and the number of dual positive cells for the RBR signals and FosB immunoreactivity within the square was counted. The total number of cells in three randomly selected sections from six sections per RBR-injected Sedentary mouse was regarded as the number of immunopositive cells. To avoid any bias in the results of the quantitative analysis, the investigators were blinded to all experimental groups throughout the quantitative analyses.

### Statistical analysis

Quantitative data are presented as mean ± standard error of the mean. A three-way repeated measures analysis of variance (ANOVA) followed by Tukey’s post-hoc test was used to compare withdrawal latency among the experimental groups. A two-way ANOVA followed by Tukey’s post-hoc test was used to compare the number of immunopositive cells among the experimental groups. The relationship between the two variables was tested using Pearson’s correlation, and the criterion for statistical significance was p < 0.05. Statistical analyses were performed using Graph Pad Prism version 6.03 (GraphPad Software).

## Data Availability

All data generated or analyzed during this study are included in this published article.
